# Spanish Adaptation of the Career Commitment Scale: Psychometric Evidence and Associations with Stress and Health Across the Lifespan

**DOI:** 10.3390/healthcare13233165

**Published:** 2025-12-03

**Authors:** Tatiane Cristine Fröelich, Carmen Moret-Tatay, Manoela Ziebell de Oliveira

**Affiliations:** 1 Doctoral School, Catholic University of Valencia San Vicente Mártir, Plaza de San Agustín, 3 Esc. A Entresuelo 1, 46001 Valencia, Spain; 2 Postgraduate Psychology Program, Pontifical Catholic University of Rio Grande do Sul, Porto Alegre 90619-900, Brazil; manoela.oliveira@pucrs.br

**Keywords:** career resilience, career planning, career identity, stress, mental health, career adaptability, Spanish, psychometric validation

## Abstract

**Introduction/Objectives:** In the context of Spain’s persistently high job insecurity and evolving labor market, understanding how individuals sustain career engagement is critical. This study aimed to adapt and validate the Career Commitment Scale (CCS) for use in the Spanish population and examine its relationship with career adaptability, mental health, and stress across different age groups. **Methods:** Using a sample of 418 participants, exploratory and confirmatory factor analyses confirmed the CCS’s original three-factor structure, career identity, planning, and resilience, with satisfactory fit indices and strong reliability. Criterion-related validity was supported through significant positive correlations with career adaptability and negative associations with depression, anxiety, and stress. Test–retest analysis over a three-month interval showed moderate-to-strong temporal stability. **Result:** CFA confirmed the factor structure. A moderation analysis revealed that stress moderated the relationship between age and career resilience: older individuals demonstrated higher resilience under low stress conditions, but this benefit diminished under high stress exposure. **Conclusions:** These findings highlight the relevance of career commitment as a multidimensional construct closely linked to mental well-being and adaptive functioning in uncertain labor markets. The validated CCS provides a reliable tool for research and practice, offering new insights into how career motivation interacts with age and psychological stress across the lifespan. This validation has meaningful implications for organizational practices, career counseling, and public policy, as career commitment can buffer against Spain’s chronic unemployment and job precarity—particularly for younger workers and those in non-standard employment.

## 1. Introduction

The Spanish labor market has undergone significant changes over the past two decades. Since the Global Financial Crisis of 2008, high long-term and youth unemployment, a substantial share of temporary employment contracts, and job insecurity distinguish Spain from other OECD economies [1], such as Germany and France [2]. As of December 2024, the country’s unemployment rate stood at 10.61% (compared to 6.3% in the euro area). Despite being the lowest rate since 2008 [3,4], it reveals the highest job insecurity among OECD nations [1], hindering economic growth and workforce competitiveness [3]. Such insecurity creates an unprecedented level of labor market duality, where workers oscillate between precarity and stability [5]. Evidence suggests that job insecurity also contributes to work-related stress and exhaustion, leading to increased health-related absences, decreased work engagement and retention, lower job satisfaction, and productivity for most individuals. However, some workers remain committed due to economic necessity and the perceived cost of leaving their jobs [5,6].

This scenario highlights the importance of critically understanding the workforce’s career commitment as its impacts are not uniform across individuals. It is a well-established fact that age significantly influences how people experience and respond to insecurity: while younger workers face entry barriers and disrupted career starts, older workers often deal with age discrimination and uncertainty surrounding retirement [1,3]. These age-specific challenges demand that workers navigate distinct obstacles. In both cases, career commitment plays a relevant role, mitigating the adverse effects of job insecurity and enabling employees to persevere despite uncertainty across the lifespan [6]. Therefore, understanding these age-differentiated responses is crucial for developing effective support strategies tailored to each age demographic.

Career commitment refers to an individual’s attitudes and willingness to maintain membership in an occupational field, or an emotional linkage between an individual and his or her occupational field [7,8]. It is distinct from organizational commitment (loyalty to the employer) and broader work commitment (career and organizational commitment, job involvement, and work ethic) [7,9,10]. While literature offers several instruments and models to assess career commitment [11,12], the three-dimensional Career Commitment Scale (CCS), proposed by Carson and Bedeian (1994) [8], is widely validated and conceptually robust. The instrument measures career identity (the extent to which individuals define themselves by their work), resilience (the persistence to remain in an occupational field, especially when facing challenges), and planning (the motivation to advance one’s career and establish career-related goals) with adequate reliability indices (α = 0.79–0.85). The CCS has been adapted to diverse cultural contexts and languages, like Brazil [13], Malaysia and Australia [14], with evidence confirming its original three-factor structure and cross-cultural potential in both collectivist and individualist cultures. Despite it being a valuable and reliable measure, in our review, we found no validated Spanish version of the CCS is available. This absence challenges the use of the available CCS version in Spain, given its peculiar labor market reality: a developed economy exhibiting a developing-economy level of precarity [1,3]. This reality creates a theoretical and measurement context fundamentally different from that of the existing adaptations.

The lack of a validated Spanish CCS limits research on how workers remain committed to their occupational field amidst Spain’s unique instability. Evidence consistently shows that career commitment enhances positive career outcomes, such as performance, motivation, retention, job crafting, job satisfaction, well-being, and career sustainability, while mitigating negative outcomes, including turnover intentions and stress [7,8,15,16,17,18,19]. Literature has also highlighted the correlation between career commitment and career adaptability, underscoring the need to understand how workers navigate dynamic and nonlinear career paths throughout the lifespan, as well as the interaction between career variables and stress [16,19,20,21,22,23,24]. Based on such evidence, we argue that without a reliable, context-specific measure, researchers and practitioners have restricted capability to develop and evaluate career counseling, workforce policies, and organizational interventions tailored to this high-precarity environment. This gap limits the understanding of how commitment functions across workers’ lifespan under Spain’s specific pressures, thereby delaying solutions that improve individual and organizational outcomes.

Career development is a lifelong, dynamic process shaped by personal, social, and environmental factors. As individuals move through life stages, their career needs, goals, and constraints shift in response to both internal changes and external demands. Modern career paths are increasingly nonlinear and transition-rich, requiring adaptability, self-reflection, and proactive planning [21]. Contemporary career theories emphasize self-directed, value-driven career navigation in a world of flexible but uncertain employment structures [22]. The increased autonomy and unpredictability associated with modern careers also contribute to psychosocial stress—particularly during transitions like job loss, career changes, or retirement planning. Stress can disrupt career decision-making and lower self-efficacy, especially in contexts lacking social or institutional support [23]. The ability to manage this stress via personal resources, like resilience, plays a critical role in maintaining career engagement and well-being over time [24], but its effectiveness may vary significantly across age groups.

Given the relationships between career commitment and key variables like adaptability and well-being and considering the significant age-differentiated impacts of Spain’s labor market conditions, a critical research gap exists: no validated Spanish version of the Career Commitment Scale (CCS) is available, despite successful adaptations in Brazil, Malaysia, and Australia [13,14]. This absence is particularly problematic given Spain’s unique position as a developed economy exhibiting developing-economy-level precarity [1,3], which makes it a labor market context fundamentally different from existing adaptations.

This gap has substantial implications for both research and practice. Theoretically, it limits our understanding of career commitment dynamics in a context characterized by unprecedented labor market duality and age-differentiated challenges. Practically, it hinders the development of evidence-based career counseling strategies, workforce policies, and organizational interventions tailored to Spain’s specific conditions. Without a psychometrically sound instrument, researchers cannot accurately measure how workers of different ages navigate this high-precarity environment, creating a significant barrier to improving individual well-being, organizational outcomes, and national competitiveness in one of Europe’s most precarious labor markets.

Therefore, this study aims to adapt a measure of career commitment for use with the Spanish population, employing both exploratory and confirmatory factor analyses to examine its underlying factor structure. Additionally, the study aims to provide criterion-related validity evidence, using career adaptability as a convergent measure and mental health as a divergent measure, ensuring a comprehensive approach to career commitment assessment in Spain. Lastly, it is essential to examine the moderating effects of stress on the relationship between career commitment and age, as stress can differentially impact career trajectories at various life stages. In this way, different Hypothesis were developed as follows:

**Hypothesis** **1.** *The Spanish adaptation of the Career Commitment Scale confirms a three-factor structure (career identity, career resilience, and career planning).*

**Hypothesis** **2.** *The Spanish adaptation of the Career Commitment Scale demonstrates convergent validity through significant positive correlations with career adaptability, and discriminant validity through weaker or negative correlations with mental health indicators, such as anxiety and stress.*

**Hypothesis** **3.** *The Spanish Career Commitment Scale shows strong test–retest reliability, reflected in high temporal stability of the subscale scores (career identity, career resilience, and career planning) across two measurement occasions.*

**Hypothesis** **4.** *Perceived stress moderates the relationship between age and career commitment dimensions, such that the effect of age on career commitment dimensions varies depending on perceived stress levels.*

## 2. Materials and Methods

### 2.1. Participants and Ethics

A total of 417 individuals volunteered to participate in the study. They were randomly divided into two subgroups. 46% were women. The mean of age was 41.74 (SD = 12.90). Most respondents (78.7%) did not indicate a secondary profession. Among those who did, approximately 8.4% reported working in education and research, including roles such as university professors, lecturers, and academic tutors. About 6.7% were engaged in private practice or freelance work—this includes psychologists, consultants, therapists, and self-employed professionals. A smaller share, around 4.1%, were involved in creative, communication, or media-related activities like writing, journalism, or public speaking. Another 3.5% reported positions in business or technical sectors, including IT, real estate, and engineering. The remaining 2.6% held various roles such as healthcare workers, salespeople, or volunteers, reflecting a wide diversity of secondary occupations.

Thus, this study used an independent sample comprising 209 participants for the exploratory factor analysis (EFA) and 208 participants for the confirmatory factor analysis (CFA). The data were collected from individuals who met the study’s inclusion criteria, ensuring a broad representation of relevant characteristics for evaluating the constructs of identity, planning, and resilience. 

The research project was approved by the Ethics Committee of the Catholic University of Valencia “Saint Vincent Martyr” (Code: UCV/2023-2024/066 on 11 January 2024) and the Ethics Committee of the Pontifical Catholic University of Rio Grande do Sul (PUCRS), Brazil (No: CAAE 75168023.1.0000.5336 on 16 November 2023). All participants were informed about the study’s objectives and provided written informed consent before participation.

### 2.2. Measures

The researchers collected data using a survey online containing sociodemographic questions to characterize the sample, and three psychometric measures. Each of them is described below.

The Career Adapt-Abilities Scale (CAAS). Was developed to assess the construct of career adaptability across various countries [25], including Spain [26]. The scale consists of 24 items, rated on a five-point Likert scale ranging from 1 (Developed Little or Not at All) to 5 (Developed Extremely Well). The CAAS conceptualizes career adaptability as a set of psychosocial resources that help individuals manage their careers effectively in dynamic and uncertain work environments. It comprises four dimensions, each with six items: Concern evaluates the ability to think ahead and prepare for future career challenges (α = 0.79; sample item: Thinking about what one’s future will be like.); Control reflects the sense of ownership and responsibility over one’s career decisions (α = 0.80; sample item: Taking responsibility for one’s own future); Curiosity comprises the motivation to explore different career paths and opportunities (α = 0.83; sample item: Exploring options before making a choice) and Confidence reflects the belief in one’s ability to overcome obstacles and succeed in career goals (α = 0.84; sample item: Overcoming obstacles).

DASS-21 (Depression, Anxiety, and Stress Scale—21 items). This is a short version of the original DASS-42 [27]. The DASS-21 consists of 21 items, with seven items per dimension, rated on a four-point Likert scale (0 = Did not apply to me at all, 3 = Applied to me very much or most of the time). It measures psychological distress across three dimensions: Depression refers to a lack of motivation, anhedonia, and feelings of worthlessness; Anxiety measures physiological hyperarousal, panic, and excessive worry and Stress evaluates difficulty in relaxation, tension, and irritability. The scale is widely used in both clinical and research settings, with solid psychometric properties across different cultural contexts, including Spain. Ruiz et al. (2017) [28] found alpha values of 0.89 for Depression, 0.85 for Anxiety, and 0.87 for Stress.

The Career Commitment Scale (CCM) was developed to assess the construct of career commitment and its relationship with various work-related outcomes [8]. The scale consists of 12 items, rated on a five-point Likert scale ranging from 1 (Strongly Disagree) to 5 (Strongly Agree), conceptualizing career commitment as an individual’s motivation to work in a chosen vocation, including aspects of career identity, planning, and resilience. It comprises three dimensions, each with four items: Career Identity reflects the degree of emotional association and identification with one’s career (α = 0.81; sample item: My line of work/career field is an important part of who I am); Career Planning assesses the extent to which individuals set career-related goals and engage in developmental strategies (α = 0.85; sample item: I have created a plan for my development in this line of work/career field); Career Resilience represents persistence in maintaining one’s career despite difficulties (α = 0.79; sample item: Though my line of work/career field has its difficulties, I continue to try hard). In the present study, we evaluated the internal consistency of three subscales (Identity, Planning, and Resilience) across both the EFA and CFA samples. In the EFA sample, reliability estimates were satisfactory to excellent: Cronbach’s alpha ranged from 0.71 (Identity) to 0.89 (Resilience), and Planning 0.82. For McDonald’s omega ranged from 0.85 (Identity) to 0.91 (Resilience), and 0.89 for Planning, indicating good internal consistency and suggesting that the items within each subscale coherently reflect their underlying constructs. In the CFA sample, a similar pattern emerged, described as follows: Identity (α = 0.64; ω = 0.83) and consistently high reliability for Planning and Resilience (α between 0.80 and 0.84; ω between 0.88 and 0.89).

### 2.3. Procedure

The translation and cultural adaptation of the Career Commitment Scale (CCS) for use in Spain followed a combination of procedures suggested by [29,30]. The process began by contacting the authors of the CCS via email to request authorization for the translation and adaptation of the instrument into Spanish. Upon receiving approval, the following steps were undertaken:

1. Two independent translators (T1 and T2), both native Spanish speakers fluent in English, produced two separate Spanish versions of the scale.
2. A third translator (T3), a psychologist with extensive expertise in English, compared both translations, discussed inconsistencies, and synthesized them into a single version.
3. An independent back-translation (T4) was conducted by a Spanish-speaking translator certified in English. The back-translated version was then compared with the original scale to verify its accuracy and conceptual equivalence.
4. Once approved, the Spanish version of the CCS was evaluated by five professionals, who assessed the clarity, relevance, and cultural appropriateness of the items. Participants provided feedback, and necessary adjustments were made before finalizing the adapted version.
5. The reviewed version was sent to the original authors of the CCS to ensure compatibility between the Spanish and original English versions.

After that, the researchers collected data from the target population. Recruitment of participants involved sending invitations via email, WhatsApp, and LinkedIn to potential participants. Those who were interested in participating in the study had to access the online survey hosted on the Qualtrics.com platform. Only after reading and agreeing to the informed consent could they complete the survey. Upon completion, all participants received a report on their results, along with tips on how to address the assessed variables in their daily lives.

### 2.4. Data Analysis

To examine the psychometric properties of the instrument, we split the data into two independent subsamples. The first subsample was used for an Exploratory Factor Analysis (EFA), and the second for a Confirmatory Factor Analysis (CFA). For the initial EFA, the maximum likelihood extraction method was used, coupled with Varimax rotation to optimize the interpretability of the factors. The suitability of the data for factor analysis was assessed using the Kaiser-Meyer-Olkin (KMO) measure of sampling adequacy and Bartlett’s test of sphericity.

The CFA was subsequently conducted using an independent sample to test the hypothesized factor structure that emerged from the EFA. The confirmatory factor analysis (CFA) employed a range of indices to assess model fit and ensure the robustness of the proposed structure. The chi-square statistic (χ^2^) and the relative chi-square (χ^2^/df) were used as primary indicators of overall model fit, with a relative chi-square value below 3 considered indicative of an acceptable model [31]. The root mean square residual (RMR) and the goodness-of-fit index (GFI) were applied to evaluate the degree to which the model replicated the observed data, with RMR values closer to 0 and GFI values above 0.90 indicating a good fit [32,33]. The adjusted goodness-of-fit index (AGFI) adjusted for model complexity, where values above 0.90 were deemed satisfactory [34]. Comparative indices such as the comparative fit index (CFI) and the incremental fit index (IFI) were employed, with values exceeding 0.95 reflecting excellent fit [35,36]. The normed fit index (NFI) was also used, where values over 0.90 indicated acceptable fit [37]. The root mean square error of approximation (RMSEA), accompanied by its confidence interval and PCLOSE value, assessed the model’s approximation to the population covariance matrix, with values below 0.06 and a PCLOSE above 0.50 considered indicators of good fit [38,39]. Parsimony-adjusted measures, including the parsimony ratio (PRATIO), the parsimony normed fit index (PNFI), and the parsimony comparative fit index (PCFI), were utilized to balance model complexity and fit, with values around 0.70 or higher suggesting a well-balanced model [40]. Lastly, the Akaike Information Criterion (AIC) was used for comparative model assessment, with lower values indicating better model parsimony [41].

Normality was evaluated by inspecting residual plots (histograms and Q–Q plots) and skewness/kurtosis statistics. Multicollinearity was assessed using variance inflation factors (VIF) and tolerance values for all predictors and interaction terms All continuous variables were standardized prior to analysis (i.e., Z-scores). In the model, age was the independent variable, psychological resilience was the dependent variable, and perceived stress exposure was the moderator. SPSS v23 (IBM) and AMOS v18 softwares were employed.

## 3. Results

First, this study conducted an exploratory factor analysis (EFA) aimed at identifying the underlying dimensions within a set of variables related to constructs of identity, planning, and resilience. The maximum likelihood extraction method with Varimax rotation was employed to enhance interpretability. Data were sourced from an independent sample of 209 cases, with listwise exclusion applied to any missing values, and 12 variables were included in the analysis. The Kaiser-Meyer-Olkin (KMO) measure of sampling adequacy was 0.833, indicating good suitability for factor analysis. Additionally, Bartlett’s test of sphericity was significant (χ^2^ = 1421.499; df = 66; *p* < 0.001), confirming the appropriateness of conducting the analysis.

Initial and extracted communalities showed that most retained variables demonstrated a substantial proportion of explained variance. Extraction values ranged from 0.205 (item 3 of the career planning dimension of the Career Commitment Scale) to 0.828 (item 3 of the resilience dimension of the Career Commitment Scale), highlighting an adequate representation of variables within the model. Three factors were extracted, explaining a total of 62.81% of the variance. The first factor accounted for 36.64% of the variance, the second factor for 16.42%, and the third for 9.74%, suggesting that the three factors represent distinct and relevant constructs.

The rotated varimax factor matrix in Table 1 indicated that the variables coherently grouped into three factors. The covariances among the factors were also reported, showing significant relationships between F1 and F2 (Estimate = 0.285, *p* < 0.001 Estimate = 0.285, *p* < 0.001) and between F1 and F3 (Estimate = 0.101, *p* = 0.037). The overall results suggest that the hypothesized model provides a good representation of the data, supporting the validity of the measurement structure. The results of this analysis indicate the presence of three clearly differentiated factors corresponding to the constructs of resilience, identity, and planning. These factors account for a substantial proportion of the variance, confirming the multidimensional structure of the evaluated variables.

Secondly, the CFA was conducted on the model provided satisfactory results with an independent sample (n = 208), demonstrating good fit indices across multiple measures and allowing to confirm Hypothesis 1. The χ^2^ statistic for the default model was 59.758, while df = 51. Therefore, χ^2^/2 = 1.17 (*p*-value = 0.188), further supporting the model’s adequacy. Fit indices included an RMR of 0.055, a GFI of 0.956, an AGFI of 0.933, and a PGFI of 0.625. These indices suggest that the model fits the data well. Comparative indices also reflected a strong model fit, with an NFI of 0.950, an RFI of 0.935, an IFI of 0.992, a TLI of 0.990, and a CFI of 0.992, all indicating a close match to the data.

The RMSEA for the model was 0.029 (90% CI: 0.000–0.055) with a PCLOSE value of 0.897, indicating an excellent fit. The AIC for the default model was 113.758, lower than that of the independence model (1212.539), further supporting the model’s parsimony. The model also demonstrated good parsimony-adjusted measures with a PRATIO of 0.773, a PNFI of 0.734, and a PCFI of 0.767.

### 3.1. Analysis of the Career Commitment Scale’s Criterion Validity

To examine the convergent and divergent validity of the three psychological constructs, identity, planning, and resilience, Pearson correlations were computed between these factors and the subscales of the Depression Anxiety Stress Scales (DASS-21), including depression, anxiety, and stress. This analysis addressed Hypothesis 2 by evaluating whether the constructs exhibited theoretically consistent associations with negative emotional states (convergent validity), while also maintaining sufficiently low correlations to support their conceptual distinctiveness (divergent validity). All DASS-21 subscales were strongly intercorrelated, with anxiety and stress showing the highest association (r = 0.75, *p* < 0.001), followed by anxiety and depression (r = 0.68, *p* < 0.001), and stress and depression (r = 0.66, *p* < 0.001), reflecting the shared variance commonly observed among negative affective states (Table 2).

As hypothesized, the three psychological strengths (identity, planning, and resilience) correlated negatively with all three DASS-21 dimensions, providing evidence of convergent validity. The strongest inverse correlations were observed between depression and identity (r = −0.25, *p* < 0.001), planning (r = −0.25, *p* < 0.001), and resilience (r = −0.29, *p* < 0.001). Similar negative associations were observed between resilience and both anxiety (r = −0.24, *p* < 0.001) and stress (r = −0.29, *p* < 0.001), indicating that individuals with higher resilience tended to report lower levels of emotional distress.

However, the magnitudes of these correlations were moderate to small, suggesting acceptable levels of divergent validity. Identity and planning showed the weakest correlations with anxiety (identity: r = −0.15, *p* = 0.004; planning: r = −0.08, *p* = 0.13) and stress (identity: r = −0.10, *p* = 0.063; planning: r = −0.08, *p* = 0.12), supporting the idea that these are distinct psychological constructs that are only modestly associated with general negative affect.

### 3.2. Test–Retest

To assess the temporal stability of the key psychological constructs, a test–retest reliability analysis was conducted on a subsample of 34 participants who completed the measures again after a 3-month interval. Pearson product–moment correlations indicated moderate-to-strong stability over time. Specifically, identity demonstrated a reliability coefficient of r = 0.603, planning exhibited a coefficient of r = 0.635, and resilience showed the highest temporal stability with r = 0.665.

#### An Analysis of Stress as a Moderator in the Relationship Between Age and Career Commitment

To examine whether the association between age and Career Commitment was moderated by perceived stress exposure, a moderation analysis was conducted over the whole data set. Three separate models were tested, one for each subfactor of Career Commitment. Only the model involving Career Resilience showed a significant moderating effect. In this way, the overall model was significant, F(3, 382) = 5.68, *p* = 0.0008, accounting for 4.3% of the variance in resilience scores (R^2^ = 0.043).

**Figure 1:**
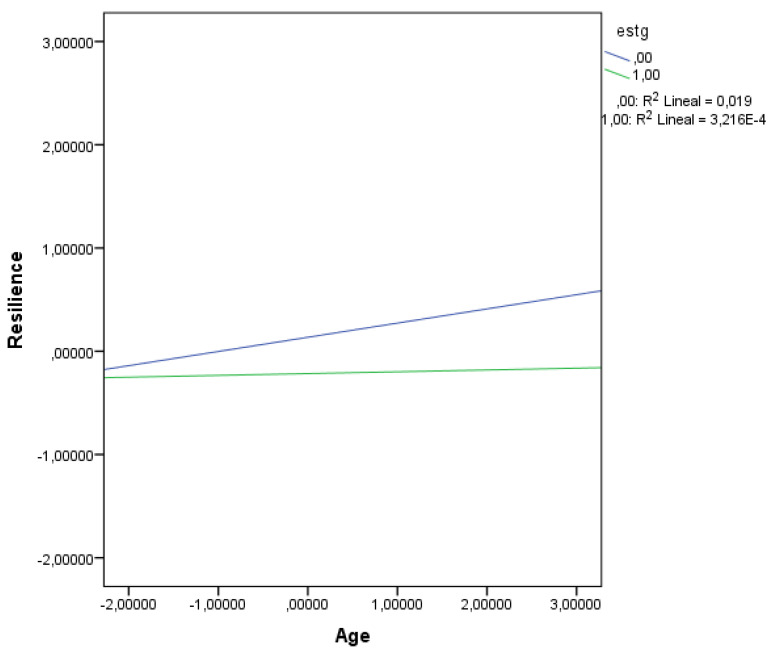
Interaction Between Age and Stress Exposure Predicting Resilience.

As depicted in Table 3, there were significant main effects of both stress exposure, B = −0.35, SE = 0.11, *p* = 0.0009, and age, B = 0.10, SE = 0.05, *p* = 0.030. However, the interaction between age and stress exposure was not statistically significant, B = −0.12, SE = 0.10, *p* = 0.245, suggesting that the effect of age on resilience did not differ significantly across levels of stress exposure. Nonetheless, conditional effects analysis showed in Table 4 revealed that at low levels of stress exposure (−1 SD), age significantly predicted greater resilience (B = 0.14, SE = 0.06, *p* = 0.016), whereas at high levels of stress exposure (+1 SD), the relationship was not significant (B = 0.02, SE = 0.09, *p* = 0.839). This interaction is also represented in Figure 1.

## 4. Discussion

The present study aimed to adapt the Career Commitment Scale into Spanish by assessing its factorial structure and exploring the interplay between career development variable and psychological stress and age. The findings strongly support the psychometric robustness of the adapted CCS and contribute to a nuanced understanding of how career commitment manifests within the Spanish labor context, a developed economy marked by high job insecurity, structural labor market duality (26.4% temporary contract vs. 11.2% OECD average), and evolving employment structures that disproportionately affect both young and older workers.

Consistent with Hypothesis 1, which aimed to confirm the underlying factor structure of the scale, results from both exploratory and confirmatory factor analyses supported a robust and stable three-factor model, comprising career identity, career planning, and career resilience, aligned with the original theoretical framework proposed by Carson and Bedeian (1994) [8]. The factor solution explained over 60% of the total variance, with strong factor loadings and satisfactory model fit indices. These results are consistent with previous validations in Brazil [13] and Malaysia [14], where the CCS has similarly demonstrated strong structural validity and cross-cultural applicability. This consistency across diverse cultural and economic contexts (from emerging economies to develop markets with structural precarity) supports the universality of the three-dimensional career commitment construct while highlighting its relevance in labor markets characterized by uncertainty.

Findings for Hypotheses 2 and 3 also demonstrated solid psychometric qualities. Internal consistency was strong across all three dimensions, and test–retest reliability showed moderate-to-high temporal stability over a three-month interval. In terms of criterion validity, the CCS dimensions negatively correlated with anxiety, depression, and stress as measured by the DASS-21, consistent with theoretical expectations [28] and prior research linking career resilience to psychological well-being [42]. These findings suggest that career identity, planning, and resilience contribute to individuals’ ability to navigate uncertain labor market conditions, reinforcing previous work linking career commitment to employability, and career sustainability [16,18], and life satisfaction [43].

The three dimensions of career commitment form an integrated protective psychological framework that is particularly salient in contexts of labor market volatility [16,43]. Career identity, which refers to the emotional and cognitive attachment to one’s vocational path, provides a stable sense of self, purpose, and direction that buffers against feelings of worthlessness and loss of control, key elements of depression [43,44]. Career planning, which is described as a proactive goal setting and future orientation, promotes self-regulatory processes that facilitate career adaptability [16,25], reduce stress by providing a sense of direction and control over one’s future, counteracting the helplessness often experienced in rapidly changing or unstable work environments [21]. Moreover, studies show that it contributes to the enhancement of emotional resilience and sustained positive mental health across the life span [24]. Career resilience, the capacity to maintain motivation and engagement despite professional challenges [45], serves as a psychological buffer that reduces emotional reactivity to occupational setbacks and mitigates depressive tendencies by fostering a sense of competence, perseverance, and hope, even when external conditions are unstable or discouraging [46,47].

When well-developed, these three dimensions collectively promote adaptive functioning [16], reduce psychological distress, and enhance overall well-being [43]. Their significance is amplified in labor markets characterized by structural insecurity, such as Spain’s, where individuals must frequently recalibrate their career paths in response to external instability. The negative correlations observed between CCS dimensions and mental health symptoms (DASS-21) underscore this protective role: individuals with stronger career identity, planning, and resilience reported lower levels of anxiety, depression, and stress, even when facing the uncertainties inherent in Spain’s dual labor market.

Lastly, a contribution of interest in this study lies in the moderation analysis addressing Hypothesis 4, which explored whether perceived stress alters the relationship between age and career resilience. According to literature, the relationship between age and career commitment is complex, exhibiting both a small linear and a more nuanced curvilinear pattern—and there is a need for more detailed investigation into the dynamic and conditional nature of age-career commitment relationships [48]. Although the interaction term was not statistically significant in our study, conditional effects indicated that age positively predicted resilience at low stress levels, but not under high stress conditions. This pattern suggests a potential attenuation of the age–resilience relationship under higher stress, although the lack of a significant interaction term limits firm conclusions. Our results relate to those of previous research showing that, for older employees, the relationship between development-focused HR practices (DHRPs) and career commitment is linear and positive—meaning that as DHRPs increase, so does commitment. In contrast, for younger employees, this relationship is inverted U-shaped: commitment increases with DHRPs up to a point, then declines if DHRPs become excessive (higher stress levels). Notably, in that study, the inverted U-shaped relationship was observed primarily among younger employees, while older employees consistently showed a positive association between DHRPs and commitment, regardless of stress level [49].

These findings align with developmental and coping theories suggesting that life experience (proxied by age) enhances resilience, but only when contextual stressors remain manageable [23]. The attenuation of age-related benefits under high stress supports Lazarus and Folkman’s (1984) [50] transactional model of stress and coping, where perceived stress can deplete cognitive-emotional resources needed for adaptive functioning. While the moderation effect was modest, it suggests that psychological resources are not fixed but context dependent. Such finding is consistent with recent work on occupational stress among Spanish professionals, where chronic exposure to stressors significantly diminished work engagement and adaptive behaviors [51].

### 4.1. Practical Implications

The validation of the CCS in Spain represents the first psychometrically validated multidimensional measure of career commitment available for Spanish-speaking populations in Europe, filling a critical gap in career assessment tools for a labor market characterized by structural duality and chronic precarity. This has meaningful implications for organizational practices, career counseling, and public policy. Career commitment can serve as a buffer against Spain’s chronically high unemployment and job precarity, particularly for younger workers and those in non-standard employment. Previous studies have shown that career commitment is positively linked to engagement, retention, and productivity [7] and may help mitigate the psychosocial toll of economic uncertainty.

Given this, organizations should invest in interventions that strengthen career planning and resilience. Tailored programs that incorporate mentoring, goal-setting workshops, and stress management training may improve employee outcomes, particularly in sectors with high turnover and temporary contracts. Public employment services could incorporate CCS assessments in vocational guidance programs, particularly for vulnerable populations transitioning into or out of employment. Identifying individuals with low career commitment scores could trigger targeted interventions to enhance career identity formation, planning skills, and resilience-building strategies. In addition, organizational factors such as career support, flexible policies, and developmental opportunities can buffer stress and enhance sustainable career paths, especially in aging or vulnerable populations [46].

### 4.2. Limitations and Future Research

First, although our sample comprised employed workers sufficient for factor analysis, we did not collect data on participants’ unemployment history, contract type (permanent/temporary), or gig economy involvement. This means our sample likely included individuals from vulnerable groups (e.g., those with prior long-term unemployment or temporary contracts), but we were unable to identify or analyze these subgroups. Consequently, our findings limit our ability to examine how career commitment functions among those most affected by Spain’s labor market duality. Future research should address our measurement limitation by explicitly collecting employment history and contract type data. Second, the reliance on self-report measures introduces potential bias, though this is common in psychological research. Future studies should incorporate multi-informant designs or behavioral assessments where feasible.

Additionally, the moderation model suggests that stress may reduce the positive influence of age on resilience, but the lack of a significant interaction term limits firm conclusions. Longitudinal or experience-sampling methodologies could provide richer insights into how stress fluctuates and interacts with developmental variables over time. Exploring other moderators such as personality traits, coping styles, or organizational climate could also yield deeper understanding. In this way, cross-national comparisons would be valuable to examine whether the CCS behaves similarly in other Southern European labor markets (e.g., Italy, Greece, Portugal) or Latin American contexts that share Spain’s structural challenges. Such studies could clarify whether the three-factor structure and criterion validity patterns observed can be generalized to other realities and Spanish-speaking populations or reflect unique features of Spain’s labor market. Moreover, linking career commitment scores with objective career outcomes (e.g., promotions, tenure, absenteeism) could expand the instrument’s applied relevance.

In sum, this study offers a rigorous adaptation of the Career Commitment Scale in Spanish, demonstrating adequate psychometric properties and utility for understanding the interplay between career motivation, psychological resilience and mental health. Career identity, planning, and resilience emerged as coherent, measurable constructs with predictive value for well-being and adaptability—particularly relevant in Spain’s context of structural labor market precarity. While age enhances resilience, high stress may erode this effect, highlighting the importance of workplace and policy interventions aimed at reducing stress and supporting career development across the lifespan. These findings underscore the CCS’s relevance in both individual counseling and broader employment strategies to build a more engaged and sustainable workforce in Spain.

## 5. Conclusions

The present study aimed to validate the Career Commitment Scale (CCS) in the Spanish context by examining its factor structure, reliability, and validity. The findings from the exploratory factor analysis (EFA) provided strong support for a three-factor structure encompassing career identity, career planning, and career resilience, aligning with the theoretical framework proposed by Carson and Bedeian (1994) [8]. The confirmatory factor analysis (CFA) further confirmed this structure, demonstrating good model fit across multiple statistical indices. Therefore, the results suggest that career commitment is a multidimensional construct, in which career identity, planning, and resilience play distinct yet interrelated roles in individuals’ career trajectories. In addition, the high standardized factor loadings and the internal consistency values reinforce the robustness of the CCS as a psychometrically sound instrument for assessing career commitment among Spanish-speaking populations. In conclusion and given Spain’s high levels of job insecurity and temporary employment, career commitment emerges as a critical psychological resource that can enhance work engagement, career adaptability, and long-term employability. The validation of the CCS in Spain—the first multidimensional measure of its kind in Spanish-speaking Europe—holds important theoretical and practical implications for organizational development, career counseling, and public employment policy aimed at supporting workers in challenging work contexts.

## Figures and Tables

**Table 1:** Unrotated and Varimax Rotated Factor Loadings after Maximum Likelihood Extraction.

Factor	Item	Mean (SD)	Factor 1	Factor 2	Factor 3	Rotated F1	Rotated F2	Rotated F3
Identity	Item 1	4.20 (0.83)	0.587	−0.063	0.47	0.261	−0.021	**0.708**
	Item 2	4.11 (0.91)	0.656	−0.008	0.557	0.258	0.032	**0.821**
	Item 3	4.16 (0.87)	0.697	0.016	0.514	0.307	0.071	**0.807**
	Item 4	3.75 (1.26)	0.257	0.164	0.283	0.024	0.163	**0.382**
Planning	Item 1	3.67 (1.03)	0.814	−0.178	−0.264	**0.849**	0.041	0.205
	Item 2	3.79 (1.16)	0.324	−0.084	−0.08	**0.329**	−0.001	0.103
	Item 3	3.48 (1.07)	0.833	−0.164	−0.366	**0.912**	0.076	0.13
	Item 4	3.39 (1.11)	0.769	−0.105	−0.159	**0.739**	0.083	0.272
Resilience	Item 1	2.61 (1.01)	0.142	0.57	−0.09	0.011	**0.594**	0.02
	Item 2	2.65 (1.18)	0.253	0.755	−0.104	0.06	**0.797**	0.074
	Item 3	2.84 (1.19)	0.271	0.767	−0.099	0.07	**0.812**	0.089
	Item 4	3.11 (1.19)	0.24	0.763	−0.064	0.028	**0.795**	0.102

Note. Extraction method: Maximum likelihood. Rotation method: Varimax with Kaiser normalization. Values retained are shown in bold.

**Table 2:** Pearson Correlations Between Psychological Strengths and DASS-21 Subscales.

Variable	Anxiety	Stress	Depression	Identity	Planning	Resilience
Anxiety	—					
Stress	0.75 **	—				
Depression	0.68 **	0.66 **	—			
Identity	−0.15 **	−0.10	−0.25 **	—		
Planning	−0.08	−0.08	−0.25 **	0.47 **	—	
Resilience	−0.24 **	−0.29 **	−0.29 **	0.24 **	0.20 **	—

Note. ** *p* < 0.01 (2-tailed). Values represent Pearson’s *r* coefficients.

**Table 3:** OLS Regression Coefficients for the Moderation Model Predicting Resilience.

Predictor	B	SE	t	*p*	95% CI
Constant	0.04	0.05	0.78	0.434	[−0.06, 0.14]
Stress (DASS-21)	−0.35	0.11	−3.34	0.001	[−0.56, −0.15]
Age	0.1	0.05	2.18	0.03	[0.01, 0.20]
Age × Stress	−0.12	0.1	−1.16	0.245	[−0.32, 0.08]

Note. B = unstandardized coefficient; SE = standard error; CI = confidence interval.

**Table 4:** Conditional Effects of Age on Resilience at Values of Stress Exposure.

Stress Exposure (estg)	B	SE	t	*p*	95% CI
−1 SD (Low)	0.14	0.06	2.42	0.016	[0.03, 0.25]
+1 SD (High)	0.02	0.09	0.2	0.839	[−0.15, 0.19]

## Data Availability

The data presented in this article are part of a doctoral thesis conducted under a cotutelle and dual-degree agreement between the Pontifical Catholic University of Rio Grande do Sul and the Universidad Católica de Valencia San Vicente Mártir. Datasets will be available after this project ends (first author).
